# Prognostic Value of Genotype–Phenotype Correlations in X-Linked Myotubular Myopathy and the Use of the Face2Gene Application as an Effective Non-Invasive Diagnostic Tool

**DOI:** 10.3390/genes14122174

**Published:** 2023-12-03

**Authors:** Katarína Kušíková, Andrea Šoltýsová, Andrej Ficek, René G. Feichtinger, Johannes A. Mayr, Martina Škopková, Daniela Gašperíková, Miriam Kolníková, Karoline Ornig, Ognian Kalev, Serge Weis, Denisa Weis

**Affiliations:** 1 Department of Pediatric Neurology, Faculty of Medicine, Comenius University Bratislava and National Institute of Children’s Diseases, 83340 Bratislava, Slovakia; katarina.marosova@gmail.com (K.K.);; 2 Department of Molecular Biology, Faculty of Natural Sciences, Comenius University, 84215 Bratislava, Slovakia; 3 Institute for Clinical and Translational Research, Biomedical Research Center, Slovak Academy of Sciences, 84505 Bratislava, Slovakia; 4 University Children’s Hospital, SalzburgerLandeskliniken (SALK), Paracelsus Medical University Salzburg, 5020 Salzburg, Austria; r.feichtinger@salk.at (R.G.F.);; 5 Department of Metabolic Disorders, Institute of Experimental Endocrinology, Biomedical Research Center, Slovak Academy of Science, 84505 Bratislava, Slovakia; 6 Division of Neuropathology, Department of Pathology and Molecular Pathology, Neuromed Campus, Kepler University Hospital, Johannes Kepler University, 4020 Linz, Austria; 7 Department of Medical Genetics, Kepler University Hospital Med Campus IV, Johannes Kepler University, 4020 Linz, Austria

**Keywords:** *MTM1* gene, myotubularin, X-linked myotubular myopathy, centronuclear myopathy, genotype–phenotype correlations, DeepGestalt technology, Face2Gene application

## Abstract

Background: X-linked myotubular myopathy (XLMTM) is a rare congenital myopathy resulting from dysfunction of the protein myotubularin encoded by the *MTM1* gene. XLMTM has a high neonatal and infantile mortality rate due to a severe myopathic phenotype and respiratory failure. However, in a minority of XLMTM cases, patients present with milder phenotypes and achieve ambulation and adulthood. Notable facial dysmorphia is also present. Methods: We investigated the genotype–phenotype correlations in newly diagnosed XLMTM patients in a patients’ cohort (previously published data plus three novel variants, *n* = 414). Based on the facial gestalt difference between XLMTM patients and unaffected controls, we investigated the use of the Face2Gene application. Results: Significant associations between severe phenotype and truncating variants (*p* < 0.001), frameshift variants (*p* < 0.001), nonsense variants (*p* = 0.006), and in/del variants (*p* = 0.036) were present. Missense variants were significantly associated with the mild and moderate phenotype (*p* < 0.001). The Face2Gene application showed a significant difference between XLMTM patients and unaffected controls (*p* = 0.001). Conclusions: Using genotype–phenotype correlations could predict the disease course in most XLMTM patients, but still with limitations. The Face2Gene application seems to be a practical, non-invasive diagnostic approach in XLMTM using the correct algorithm.

## 1. Introduction

Myotubularin (MTM1), a protein of 603 amino acids, is a member of the myotubularin superfamily. It functions as an endosomal lipid phosphatase, which acts, for example, on phosphatidylinositol 3-phosphate, a crucial lipid in the intracellular signaling pathway. It regulates intracellular membrane trafficking and vesicular transport processes, particularly in the myocytes of skeletal muscles [1,2,3]. Myotubularin constitutes four conserved functional domains: (1) PH-GRAM (Pleckstrin Homology-Glucosyltransferase, Rab-like GTPase Activator, and Myotubularin) from amino acid 29 to 160 of MTM1, which plays a crucial role in targeting the various myotubularins to a specific intracellular compartment; (2) RID (Rac1-Induced recruitment Domain) from position 161 to 272, which is unnecessary in the recruitment of the MTM1 protein to the plasma membrane; (3) PTP/DSP (Protein Tyrosine Phosphatase/Dual-Specificity Phosphatase) from position 273 to 471, which dephosphorylates phosphatidylinositol 3-phosphate (PtdIns3P) and phosphatidylinositol 3,5-bisphosphate (PtdIns(3,5)P2) into phosphatidylinositol (PtdIns) and phosphatidylinositol 5-phosphate (PtdIns5P), respectively; and (4) SID (SET-protein Interaction Domain) from position 435 to 486, where interaction between the SID domain of myotubularins and proteins with a SET domain could regulate the expression of some genes. These four functional domains of the MTM1 protein are followed by a C-terminal coiled-coil motif and a PDZ binding domain [3]. Myotubularin is encoded by the *MTM1* gene (MIM * 300415, gene locus Xq28), composed of 15 exons [4], while the start codon is located in exon 2. To date, more than 590 variants (LOVD database https://www.lovd.nl/ (accessed on 1 November 2023), from which more than 530 are classified as pathogenic and likely pathogenic) have been found in the *MTM1* gene, distributed throughout the *MTM1* gene, with some recurrent variants but no unambiguous hotspots in the gene.

Mutations in the *MTM1* gene lead to congenital myopathy called X-linked myotubular myopathy (XLMTM, MIM # 310400, ORPHA: 596) as a subtype of centronuclear myopathies group, first described in 1966 by Spiro et al. [5]. XLMTM is inherited in an X-linked recessive manner, and its prevalence is 1:50,000 in males [6]. Three clinical forms of XLMTM have been classified as severe, moderate, and mild according to the severity of the disease course: achieving the milestones, ambulation, and ventilatory support needed [7,8,9]. In most cases (more than 80%), XLMTM presents in the neonatal period, typically immediately after birth, by severe global hypotonia (floppy infant), hypo- to areflexia, and different degrees of respiratory insufficiency. Weak fetal movements and polyhydramnios can be present even prenatally. Miscarriages or stillbirths also occur. In early infancy (in the first year of life), 25% of patients die due to respiratory failure [10], and 48% of patients die by 18 months of age [8]. However, some XLMTM patients achieve respiratory and ambulatory independence and adulthood. Gene therapy in XLMTM is under investigation, and symptomatic treatment remains the standard [11]; early diagnosis is therefore essential.

The majority of XLMTM patients have characteristic facial features (myopathic facies, uni- or bilateral ptosis, dolichocephaly with a high forehead and long face, midface hypoplasia, and a narrow, high-arched palate with malocclusion), becoming more pronounced with age, a birth length above the 97th percentile, long hands, feet, and fingers, as well as a rare condition called peliosis hepatis in up to 5% of individuals [7,10,11,12]. Thanks to the typical histopathological picture, relatively low costs, and quick results, invasive muscle biopsy has, for a long time, been considered a standard diagnostic method. However, the innovative non-invasive approach of applying DeepGestalt technology to facial features in the diagnosis of XLMTM has not yet been investigated.

As mentioned, some XLMTM patients achieve respiratory and ambulatory independence and adulthood, contrasting with high neonatal and infantile mortality rates. This phenotype variability logically led to questions about genotype and resulting phenotype. Several studies focusing on evaluating genotype–phenotype correlations have been published [8,9,13,14], but these correlations remain unclear.

Our study aimed to introduce a new perspective on genotype–phenotype correlations and reconsider their predictive value in the XLMTM disease course based on data obtained from databases published over more than 20 years, supported by statistical processing, as well as expansion of the *MTM1* mutational spectrum by three novel disease-causing variants identified in our patients. Considering the typical facial features, using DeepGestalt technology [15] via the Face2Gene application (FDNA, Inc., Boston, MA, USA), we aimed to determine its relevance in a non-invasive diagnostic approach in XLMTM patients, and whether it has a potential to replace the invasive muscle biopsy as the investigation supplementary to genetic testing in the future.

## 2. Subjects and Methods

### 2.1. Subjects Involved in the Statistical Analysis

**Figure 1:**
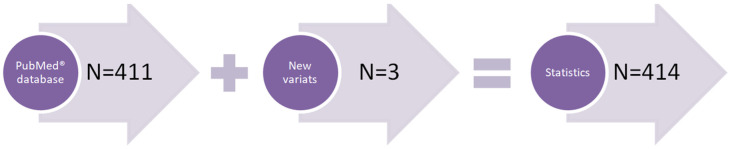
Genotype–phenotype correlations: methodologic flow chart. We included N = 411 already published cases (index patients with defined genotype and phenotype) and N = 3 novel variants from our in-house database with defined phenotypes. All index cases (N = 414) were statistically processed using Fisher’s exact test.

For the selection of subjects involved in the present analysis, we used already published data obtained retrospectively from the PubMed^®^ database, searched using the following keywords: myotubularin, *MTM1* gene, X-linked myotubular myopathy, XLMTM, X-linked centronuclear myopathy (XLCNM), and genotype–phenotype correlations in XLMTM/XLCNM (*n* = 411). In our study, we included only index male subjects (from unrelated families), with disease-causing variants identified in the *MTM1* gene and specified XLMTM phenotype (see Section 2.2 below). Data were expanded by three novel *MTM1* variants detected in patients P1–P4 from our in-house database (Figure 1).

### 2.2. Phenotype Severity

All involved subjects had a specified XLMTM phenotype, classified according to [7,8,9] into three groups, presented as: (1) “S”—severe (classic) phenotype with characteristic facial features, long-term ventilator dependence more than 12 h per day, delayed gross motor milestones, no independent ambulation, and high mortality rate in infancy; (2) “Mo”—moderate (intermediate) phenotype with less severe motor delay, prolonged periods of decreased ventilation support, at least 6 to 8 h per day without ventilatory support [7], or more than 12 h without ventilator support per day [9]; and (3) “Mi”—mild phenotype with minimal motor delay, and the lack of a need for mechanical ventilation, independent spontaneous respiratory function beyond the newborn period, and no or limited impact of the life span. Cases collected by Laport et al. (2000) were classified only into two groups: (1) “S”—severe subjects with classic phenotype; and (2) “M”—mild or moderate subjects who are long-term survivors without heavy ventilator assistance [16]. This fact was taken into account during statistical processing.

### 2.3. Variant Evaluation

Variants reported in already published XLMTM patients were considered as disease-causing and missense variants were tested in silico using the PredictSNP tool (combination of MAPP, PhD-SNP, PolyPhen-1, PolyPhen-2, SIFT, and SNAP tools) [17] to prove their pathogenicity. Classification of the three novel *MTM1* variants (from our in-house database) was performedfollowingthe consensus recommendations of the American College of Medical Genetics [18]. All collected variants were grouped according to their location in the *MTM1* gene (in the exon or intron and MTM1-specific functional domain: PH-GRAM, RID, PTP/DSP, and SID), variant type, and their impact on the resulting protein (truncating or non-truncating), and predicted to escape or undergo nonsense-mediated mRNA decay.

### 2.4. Facial Gestalt Analysis

We used DeepGestalt technology [15] via the Face2Gene application (FDNA, Inc., Boston, MA, USA), for facial dysmorphology comparison. In the facial gestalt analysis, we only enrolled patients with appropriate photo quality. Each cohort had to comprise at least ten photos. We selected four patient cohorts: (1) a cohort of XLMTM patients (14 patients); (2) a cohort of Myotonic dystrophy type 1 (MD1) patients, whose facial features, in our opinion, were most similar to XLMTM (10 patients); (3) a cohort of overall neuromuscular disorders (NMD patients: other congenital myopathies and muscle dystrophies, except XLMTM patients, 21 patients in total); and (4) a cohort of unaffected controls (11 patients). Healthy controls showed no obvious dysmorphia and were never suspected of 0having any genetic syndrome. We compared all cohorts with each other and the cohort of 11 healthy individuals in a binary comparison and as composite photos. A mean area under the curve (AUC) value was generated in the binary comparison, representing the degree of discrimination between the cohorts. The mean AUC ranged between 0 and 1, where 0 means incorrectly classified cohorts, 0.5 indicates random classifications, and 1 represents perfect separation between the cohorts. The *p*-value describes the accuracy of the binary comparison; *p* < 0.05 was considered statistically significant, indicating that the Face2Gene application can distinguish between the two cohorts.

### 2.5. Statistical Analysis of Genotype–Phenotype Correlations

For statistical evaluation, we used IBM SPSS version 28.0.1 Statistics software. For genotype–phenotype correlation: phenotype vs. protein truncation, phenotype vs. mutation type, phenotype vs. MTM1-specific functional domain, phenotype vs. nonsense-mediated mRNA decay, i.e., categorical variables, we used Fisher’s exact test.

## 3. Results

### 3.1. Novel Variants in the MTM1 Gene

**Figure 2:**
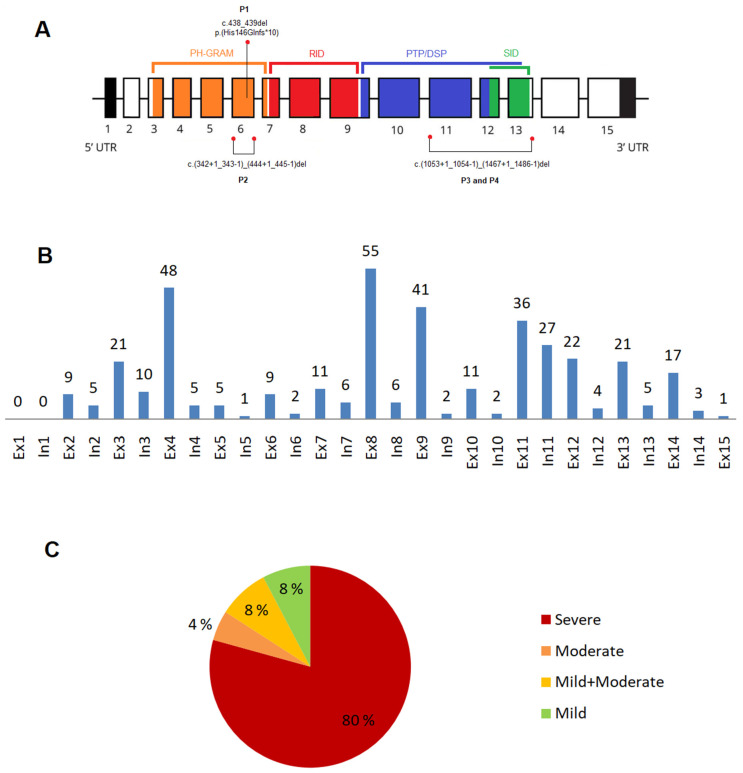
Gene structure, variant distribution, and phenotype severity proportion. (**A**) *MTM1* gene: gene structure and three novel variants identified in Patients 1–4 (P1–P4); (**B**) distribution of the disease-causing variants: *n* = 385 variants (except large deletions, *n* = 29) within the *MTM1* gene; (**C**) phenotype severity proportion: (*n* = 414). Abbreviations: In—intron, Ex—exon.

We identified three novel *MTM1* variants in our patients P1–P3 (Figure 2A). All patients presented a severe (classic) XLMTM phenotype. The hemizygous frameshift variant c.438_439delCA (p.His146Glnfs*10) in exon 6, found in P1, was evaluated as pathogenic. The hemizygous variant c.(342+1_343-1)_(444+1_445-1)del; p.(Asp115_Leu148del), which represents an in-frame exon 6 deletion, detected in P2, and the hemizygous variant c.(1053+1_1054-1)_(1467+1_1468-1)del; p.(Leu352_Gln489del), which represents an in-frame deletion of exons 11–13, found in P3 and his brother P4, were classified as likely pathogenic (Table 1). In P2 and P3, the pathogenicity of these variants was proved by histopathologic and immunohistochemical evaluation of the vastus lateralis muscle biopsy sample (for more details, see the Supplementary Materials).

### 3.2. XLMTM Cohort: Variants Type and Their Distribution

We collected 414 index cases (411 published cases so far and 3 novel variants from our in-house database); 192 variants (78.4%) in the *MTM1* gene occurred only once, whereas 53 variants (21.6%) occurred twice or more. All collected variants are summarized in the Supplementary Table S1.

#### 3.2.1. Exonic and Intronic Variants

Of 385 variants (except large deletions *n* = 29), 307 variants (79.7%) were located in exons, and 78 (20.3%) were situated in introns. More than half (53.7%) were located in exon 8 (*n* = 55; 14.3%), exon 4 (*n* = 48; 12.5%), exon 9 (*n* = 41; 10.6%), exon 11 (*n* = 36; 9.3%), and intron 11 (*n* = 27; 7%). Among other represented exons are: exon 12 (*n* = 22; 5.7%), exons 3 and 13 (each *n* = 21; 5.4%), and exon 14 and 11 (each *n* = 17; 4.4%) (Figure 2B).

#### 3.2.2. Variants in the *MTM1*-Specific Functional Domain

Most variants were located in the MTM1-specific functional domain (*n* = 276, 66.7%): 100 variants (24.1%) were located in the RID domain; 85 variants (20.5%) in the PH-GRAM domain; 64 variants (15.5%) in the PTP/DSP domain; 15 variants (3.4%) in the SID domain; and 12 variants (2.9%) in both the PTP/DSP and SIDdomains. In total, 138 variants (33.3%) were located outside of the specific domain.

#### 3.2.3. Truncating Effect and Nonsense-Mediated mRNA Decay

A total of 188 variants (45.4%) were identified as truncating and 133 (32.1%) were categorized as non-truncating. For 93 variants it was not possible to define final protein truncation. Nonsense-mediated mRNA decay was associated with 129 variants (31.2%), in 24 not (5.8%).

#### 3.2.4. Phenotype Severity and Variant Types

The severe phenotype was present in 330 (79.7%) subjects, moderate in 19 (4.6%), mild in 32 (7.7%), and a combination of mild and moderate phenotype in 33 (8%) subjects (Figure 2C). Of 414 variants, the most frequent variants were missense (*n* = 137, 33.1%), frameshift (*n* = 85, 20.5%), and nonsense variants (*n* = 70, 16.9%). Of the 137 missense variants, 87 (63.5%) were located in a functional domain, as follows: PH-GRAM 15 variants (17.2%), RID 42 variants (48.3%), PTP/DSP 25 variants (28.7%), PTP/DSP/SID 4 variants (4.6%), and SID 1 variant (1.2%). Of all 137 missense variants, 75 variants (54.7%) were associated with the severe phenotype, and the remaining (*n*= 62; 45.3%) with the mild to moderate phenotype. Less common were intronic variants (*n* = 45, 10.9%), splicing variants (*n* = 32, 7.7%), large deletions (*n* = 29, 7.0%), in/del variants (*n* = 13, 3.1%), variants affecting codon initiation (*n* = 2, 0.5%), and silent variants (*n* = 1, 0.2%). 209 variants (63.5%) associated with severe phenotype were located in the specific domain: PH-GRAM 63 variants (30.1%), RID 71 variants (34%), PTP/DSP 50 variants (23.9%), PTP/DSP/SID 11 variants (5.3%), and SID 14 variants (6.7%).

#### 3.2.5. Recurrent Variants in the *MTM1* Gene

The most frequent variants were c.1261-10A>G in intron 11 (*n* = 26), missense variant c.721C>T, p.(Arg241Cys) in exon 9 and the RID domain (*n* = 13), missense variant c.205C>T, p.(Arg69Cys) in exon 4 and the PH-GRAM domain (*n* = 12), frameshift variant c.141_144del, p.(Glu48Leufs*24) in exon 4 and the PH-GRAM domain (*n* = 10); nonsense variant c.109C>T, p.(Arg37*) in exon 3 and the PH-GRAM domain (*n* = 9), missense variant c.614C>T, p.(Pro205Leu) in exon 8 and the RID domain (*n* = 9), and missense variant c.1262G>A, p.(Arg421Gln) in exon 12 and the PTP/DSP domain (*n* = 8).

#### 3.2.6. Inter-Individual Phenotype Variability

In the cohort of 414 unrelated subjects, we found 53 variants (21.6%) occurring more than once. In 13 variants, we observed inter-individual variability, where phenotype varied from mild to severe in the case of the same mutation (Table 2).

### 3.3. Genotype–Phenotype Correlations

**Figure 3:**
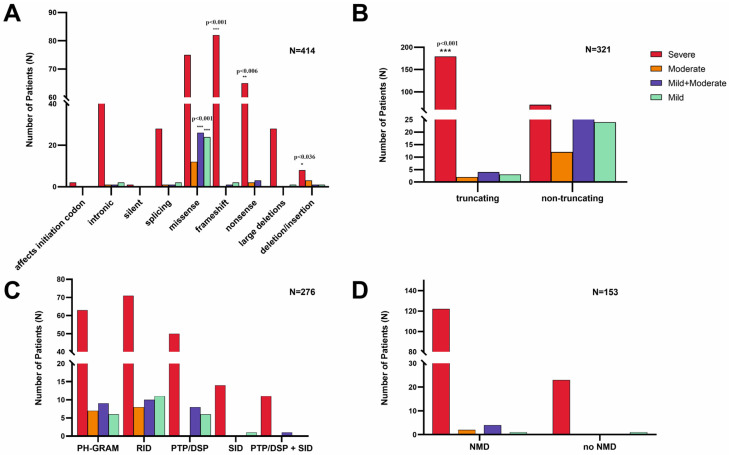
Genotype–phenotype correlations in XLMTM. (**A**) Type of disease-causing variant in the *MTM1* gene vs. phenotype: frameshift, nonsense, and in/del variants are significantly associated with a severe phenotype, as well as missense variants significantly correlating with the mild or moderate phenotype; (**B**) truncation vs. phenotype: truncating variants are significantly associated with the severe phenotype; (**C**) MTM1-specific domain vs. phenotype: there is no significant difference between specific domain and phenotype severity; and (**D**) nonsense-mediated mRNA decay (NMD) vs. phenotype: there is no significant difference between nonsense-mediated mRNA decay and phenotype severity.

For genotype–phenotype correlations, we used Fisher’s exact test. We found that 95.2% of all truncating variants were associated with severe phenotype; this association was highly significant (*p* < 0.001). Focusing on variant type vs. phenotype, we found that frameshift variants were significantly associated with the severe phenotype (*p* < 0.001), as well as nonsense variants (*p* = 0.006) and deletions/insertions (*p* = 0.036). On the other hand, missense variants were significantly associated with the mild and moderate phenotype (*p* < 0.001) compared with the other variants vs. the mild and moderate phenotype. We found no significant association between phenotype and MTM1-specific functional domain, as well as phenotype and nonsense-mediated mRNA decay (Figure 3A–D).

### 3.4. Facial Gestalt Analysis

**Figure 4:**
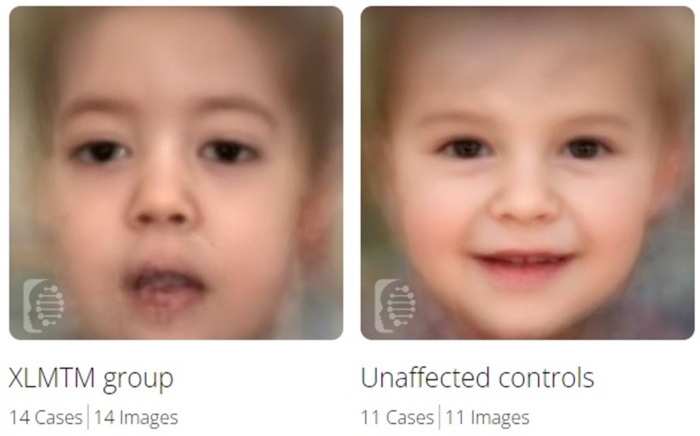
Composite images from Face2Gene for XLMTM patients and healthy controls.

Face2Gene software identified significant differences between XLMTM patients and unaffected controls (*p* = 0.001) (Figure 4) and between unaffected controls and NMD patients (*p* = 0.023). Some differences in facial gestalt could also be observe between XLMTM and MD1 patients and XLMTM patients and other NMD patients, but these were not statistically significant (see Table 3).

## 4. Discussion

X-linked myotubular myopathy (XLMTM) is a rare neuromuscular disorder associated with a high rate of neonatal and infantile mortality in patients by 18 months of age (approximately46% of all XLMTM cases) based on the severe myopathic phenotype [8]. In our opinion, the percentage of mortality is underestimated because not all cases of abortion, stillbirth, or early death of newborns are genetically investigated in the sense of congenital myopathy. For that reason, and due to the high risk of recurrence in the family, the early recognition of XLMTM is essential. On the other hand, the possible prediction of the prognosis and further disease course in newly diagnosed XLMTM patients seems to be a contributing factor; genotype–phenotype correlations could be one of these non-invasive prognostic tools.

Our study is the most extensive genotype–phenotype comparison in XLMTM, and expands the spectrum of known variants in the *MTM1* gene. Using the PubMed^®^ database plus three novel variants, we selected an XLMTM cohort of 414 index subjects with 245 different disease-causing variants. All subjects involved were males from unrelated families. Female subjects were excluded due to different disease manifestations resulting from X-linked inheritance (asymptomatic or milder phenotype in females, resulting not only from mutation type, but also due to different degrees of skewed X chromosome inactivation) [21,32].

In our study, the majority of variants in the *MTM1* gene were associated with the severe (classic) form of XLMTM (79.7%), following already published data [8,10]. According to the previously published studies focusing on phenotype correlations with the type of variant (truncating vs. non-truncating) [8], it seems that truncating variants as nonsense, frameshift, and large deletions almost always lead to severe (classic) XLMTM phenotype, while non-truncating variants such as splice site, intronic variants, as well as missense variants are associated with the milder phenotype in the minority of cases [9,13,14]. These studies were based on data from cohorts of *n* = 116 males [8] and *n* = 146 males [9]. According to the data from the *n* = 414 male subjects involved in our study, we can assume that truncating variants as frameshift, nonsense, and in/del variants are significantly associated with the severe phenotype. Missense variants are significantly associated with the moderate or mild phenotype. Thanks to this finding, we can suppose better outcomes in individuals affected by missense variants.

In our cohort, missense variants are the most common changes in the *MTM1* gene (*n* = 137, 33.1%). According to Oliveira et al. (2013), who centered their analysis on missense variants using data from the LOVD database, it was suggested that missense variants are not randomly scattered in the protein because 96% of them are located in myotubularin regions with a known function (i.e., functional domains), in contrast to 59% of nonsense variants [9]. In our study, only 63.5% (*n* = 87/137) of missense variants and 82.8% of nonsense variants (*n* = 58/70) were located in the specific functional domains, which, in the case of missense variants, corresponds to a sequence of functional domains, i.e., 71% of the length of the total MTM1 protein sequence [9]. We conclude a random scatter of the missense variants in the MTM1 protein.

Large deletions are, in almost all cases, associated with severe phenotypes. One exception in our dataset represents the deletion of the entire exon 15 reported by Tanner et al. (1999), associated with the mild XLMTM phenotype. This phenomenon could be explained by the fact that exon 15 is located at the end of the *MTM1* gene, representing less vital regions of the gene [29]. This observation could be supported by other mild phenotype frameshift variants in exon 15 [7]. No other pathogenic variants in exon 15 have been found (LOVD database https://www.lovd.nl/ (accessed on 1 November 2023)). In conclusion, large deletions, except for the isolated deletion of exon 15, are associated with a severe phenotype in all cases.

According to [9,13,14], domain-specific correlations show that mutations in PTP/DSP, SET, and RID domains are almost always associated with severe (classic) phenotypes. However, variants outside these domains are more likely to be associated with a moderate or mild phenotype. Pathogenic variants in PTP/DSP domains should be significantly associated with severe phenotypes due to the crucial enzyme activity (dephosphorylation of PtdIns3P and PtdIns(3,5)P2 into PtdIns and PtdIns5P, respectively). However, our study did not find any significant association between phenotype and the MTM1-specific functional domain, nor between phenotype and nonsense-mediated mRNA decay.

To date, more than 590 variants (LOVD database https://www.lovd.nl/ (accessed on 1 November 2023), from which more than 530 are classified as pathogenic and likely pathogenic) have been found in the *MTM1* gene. Considering that the variants detected in the *MTM1* gene are, in a high percentage, pathogenic or likely pathogenic (more than 90%), it can be assumed that every patient in whom a variant in the *MTM1* gene is detected deserves special attention, and detailed clinical evaluation in the context of XLMTM in males as well as in female probands is appropriate.

We found that from 385 point disease-causing variants in the *MTM1* gene, more than half (53.7%) are located in exon 8, exon 4, exon 9, exon 11, and intron 11. This high number of pathogenic variants found in these exons could be explained by their length without other known explanations or correlation. Some variants in the *MTM1* gene occur more than once. Tsai et al. (2005) described a potential hotspot: the recurrent pathogenic intronic variant c.1261-10A>G in a Japanese XLMTM cohort [22]. In the present cohort, we observed this variant 26 times, and according to our findings, it is the most frequently occurring variant in the *MTM1* gene, in all cases associated with the severe (classic) XLMTM phenotype.

Inter-individual variability (phenotype from classic to mild form), as well as between members of the same family, have previously been described: two families were diagnosed according to clinical examination and the histopathological findings in the biopsied muscle of affected individuals [33] in the three-generation family with variant c.540T>G, p.(Asn180Lys) [23], and a very mild phenotype in the grandfather with pathogenic variant c.1210G>A, p.(Glu404Lys), with the more severe phenotype in his grandson [34]. Bertazzi et al. (2014) described patients’ disease-causing variants affecting the PH-GRAM domain and their association with XLMTM phenotype severity [3]. While the variant p.(Val49Phe) was associated with a two-fold decrease in phosphatase activity, p.(Arg69Cys) displays wild-type phosphatase activity [35]. Our data show that the first patient is associated with the severe phenotype [24], but we collected 12 subjects with variant p.(Arg69Cys) with phenotypes varying from mild to severe. What causes inter-individual and intra-familial phenotype differences in XLMTM has yet to be discovered. We can only presume environmental factors, epigenetic factors, gene interactions, gene expression, DNA methylation, or some unknown mechanisms.

In the era of massive development and the availability of non-invasive genetic methods, the question of using invasive procedures such as muscle biopsy to diagnose neuromuscular diseases remains controversial. Based on muscle biopsy findings, some authors have suggested non-genetic starting points for determining the phenotype. Other studies, such as McEntagart et al. (2002), aimed to determine whether the level of myotubularin expression correlates with XLMTM phenotype [8]. Pierson et al. (2007) described that myofiber size correlated with *MTM1* mutation type and patient outcome [36]. According to Bryen et al. (2021), all procedures considering clinical scoring/phenotype, genetics, and muscle biopsy are equally crucial in the diagnostics of XLMTM [37]. Despite the constant improvements in harmless genetic diagnostic methods, we consider muscle biopsy, regardless of its invasiveness, to be an essential diagnostic tool in diagnosing neuromuscular diseases, especially in cases with rapid progression. Furthermore, muscle tissue can be used for possible functional studies and variant pathogenicity proof.

Considering the typical facial features in XLMTM, using DeepGestalt technology [15] via the Face2Gene application (FDNA, Inc., Boston, MA, USA), we aimed to determine its relevance in a non-invasive diagnostic approach in XLMTM patients. The Face2Gene software identified significant differences between XLMTM patients and unaffected controls, and between unaffected controls and NMD patients. Some differences in facial gestalt could also be seen between XLMTM and MD1 patients and XLMTM patients and other NMD patients, but these were not statistically significant. This could be explained by myopathic facial features commonly present in most myopathic patients. Even when patients with MDI or other NMDs are clinically entirely different, it is possible that the myopathic facial expression does not enable DeepGestalt technology to detect these features in a high-quality pattern. Therefore, DeepGestalt has limitations in distinguishing specific types of myopathic syndromes. In the future, we plan to compare XLMTM and other dysmorphic syndromes. Currently, XLMTM does not appear as one of the syndromes in the RARE tab with a composite image, meaning that the DeepGestalt algorithm (vs. DG 22.3.0) cannot yet discern it from other possible syndromes. Therefore, the second algorithm, GestaltMatcher, based on facial gestalt comparison, available in the ultra-rare tab [27], will need to be used for these cases. Based on our findings, the Face2Gene application is a potential diagnostic tool in XLMTM.

## 5. Conclusions

The presented genotype–phenotype correlations show that truncating variants such as frameshift, nonsense, and in/del variants are significantly associated with a severe phenotype. In contrast, missense variants lead, significantly in nearly half of the patients, to mild and moderate phenotypes. No significant association was found between phenotype and MTM1-specific functional domains, nor between phenotype and nonsense-mediated mRNA decay. Other associations are suggestive but are statistically not significant. Interindividual phenotypic variability, even between members of the same family, is a known phenomenon in XLMTM, which needs further investigation. Thus, the genotype–phenotype correlations are helpful but still need to be clarified and are, as a single approach, insufficient for predicting the disease course in XLMTM patients. Despite the increasing availability and effectiveness of molecular-genetic methods, muscle biopsy remains an irreplaceable, albeit invasive, method in diagnosing congenital myopathies, including XLMTM. Based on our findings, Face2Gene represents a promising non-invasive diagnostic tool for XLMTM when using the correct algorithm. However, the idea that this innovative non-invasive approach, including the Face2Gene application and the result of molecular genetic testing, will prospectively supplement an invasive muscle biopsy, is now closer to reality.

## Figures and Tables

**Table 1:** Three novel variants in the *MTM1* gene.

Patient ID	Ancestry (Ethnicity)	*MTM1* Variant	Type	Exon	Domain	ACMG Classification	Origin	Phenotype
**P1/F1** **M**	Slovakia (Caucasian)	c.438_439delCA (p.His146Glnfs*10)	Frameshift	ex6	PH-GRAM	Pathogenic	Maternal	Severe
**P2/F2** **M**	Austria (Caucasian)	c.(342+1_343-1)_(444+1_445-1)del p.(Asp115_Leu148del)	In-frame deletion	ex6	PH-GRAM	Likely pathogenic	Maternal	Severe
**P3/F3** **M**	Austria (Caucasian)	c.(1053+1_1054-1)_(1467+1_1468-1)del; p.(Leu352_Gln489)del	In-frame deletion	ex11-13	PTP/DSP-SID	Likely pathogenic	Maternal	Severe
**P4/F3** **M**	Austria (Caucasian)	c.(1053+1_1054-1)_(1467+1_1468-1)del; p.(Leu352_Gln489)del	In-frame deletion	ex11-13	PTP/DSP-SID	Likely pathogenic	Maternal	Severe

**Abbreviations: P**—patient, **F**—family, **M**—male, **ex**—exon.

**Table 2:** Recurrent disease-causing variants in *MTM1* gene with inter-individual phenotype variability in the XLMTM cohort (*n* = 414).

Variant	Type	Exon/intron	Domain	Count	Phenotype	Reference
c.98_103del, p.(Glu33_Ala34del)	Deletion	ex3	PH-GRAM	2	1S/1Mi	[19,20]
c.109C>T, p.(Arg37 *)	Nonsense	ex3	PH-GRAM	9	8S/1Mo	[21,22,23,24,25,26]
c.139_141del, p.(Lys47del)	Deletion	ex4	PH-GRAM	5	4S/1Mo	[23,25,27,28]
c.142G>T, p.(Glu48 *)	Nonsense	ex4	PH-GRAM	2	1S/1Mo	[7,29]
c.205C>T, p.(Arg69Cys)	Missense	ex4	PH-GRAM	12	2S/1Mo/6M/3Mi	[16,19,20,24,30]
c.232-26_232-23del	Intronic	in4	-	2	1S/1Mo	[19,31]
c.590C>T, p.(Thr197Ile)	Missense	ex8	RID	3	1S/1Mo/1Mi	[16,20]
c.614C>T, p.(Pro205Leu)	Missense	ex8	RID	9	8S/1Mi	[19,22,23,24,29]
c.679G>A, p.(Val227Met)	Missense	ex9	RID	3	1S/1M/1Mi	[16,19,24]
c.695A>G, p.(His232Arg)	Missense	ex9	RID	2	1S/1Mo	[19,29]
c.721C>T, p.(Arg241Cys)	Missense	ex9	RID	13	3S/1Mo/5M/4Mi	[16,19,25,26,28,29]
c.1262G>A, p.(Arg421Gln)	Missense	ex12	PTP/DSP	8	7S/1M	[16,23,24,25,30]
c.1558C>T, p.(Arg520 *)	Nonsense	ex14	-	3	1S/2M	[16,18]

**Abbreviations: in**—intron, **ex**—exon, S—severe, **Mo**—moderate, **M**—mild and moderate [16], **Mi**—mild. * stop codon.

**Table 3:** Binary comparison of the facial features of XLMTM patients, MD1 patients, NMDs patients, and healthy controls using the Face2Gene application.

Binary Comparison	No. of Cases	Mean AUC	AUC SD	*p* Value for AUC
**XLMTM vs. Unaffected**	14 vs. 11	1.00	0.01	0.001
**XLMTM vs. MD1**	14 vs. 10	0.88	0.07	0.074
**XLMTM vs. NMDs**	14 vs. 11	0.63	0.09	0.229
**Unaffected vs. NMDs**	11 vs. 11	0.99	0.02	0.023

**Abbreviations: AUC:** area under the curve, **SD:** standard deviation, **XLMTM:** a cohort of X-linked myotubular myopathy patients, **MD1:** a cohort of patients with myotonic dystrophy type 1, **NMDs:** a cohort of overall patients with neuromuscular disorder except XLMTM. A *p*-value < 0.05 represents a high degree of discrimination.

## Data Availability

All data are available in the article or in the Supplementary Materials. Genetic variants (three novel variants) reported in this study have been submitted to ClinVar, and are accessible at: https://www.ncbi.nlm.nih.gov/clinvar/ (accessed on 1 November 2023).

## Supplementary Materials

The following supporting information can be downloaded at: https://www.mdpi.com/article/10.3390/genes14122174/s1, Supplementary Materials (Case Reports including Figures S1–S4 and additional Methods and Results) and Supplementary Table S1 (Excel table, Sheet 1–2) [38,39,40,41,42].
