# Supplementary material for: Prognostic Value of Genotype–Phenotype Correlations in X-Linked Myotubular Myopathy and the Use of the Face2Gene Application as an Effective Non-Invasive Diagnostic Tool
Source: Genes (Basel). 2023 Dec 3;14(12):2174. doi: 10.3390/genes14122174 (PMC10742680; doi:10.3390/genes14122174)
Supplement: Supplementary file 1 [file genes-14-02174-s001.zip › Supplementary Data_proofreading.pdf]

Supplementary Materials

1. Case Reports

1.1. Patient 1 (P1)

Patient P1 is a Slovakian (Caucasian) male child of nonconsanguineous parents from the mother's second pregnancy (P1 has one older healthy brother). The mother mentioned weak fetal movements during pregnancy; there were no changes in amniotic fluid amount. P1 was born in the 38th week of gestation by acute cesarean section due to an alteration of heart sounds in the fetus. Birth weight was 2,050 g (Fenton: < 3rd percentile), and body length was 49 cm (Fenton: 50th percentile). Severe global hypotonia (floppy infant) and no spontaneous respiratory activity were present immediately after birth. The Apgar score was 1 (in the 1st minute), 3 (in the 5th minute) and 7 (in the 10th minute). The patient developed severe dyspnea. He had to be intubated and needed artificial ventilation support. The neurologic assessment stated pseudobulbar syndrome, the absence of reflexes, except for the neonatal primitive grip reflex, which was weak. Electromyography was without signs of myogenic or neurogenic lesion, without any signs of pathology of the neuromuscular junction. Electroencephalography was normal. Nootropic treatment was without any effect. Cardiologic examination with echocardiography was without signs of cardiomyopathy or congenital heart defects. Aphonia, weak cry, marfanoid habitus (long and narrow figure, gothic palate, pectus excavatum, long fingers of the hands and toes), cryptorchidism, fatigue appearance, bilateral ptosis, dolichocephaly, long narrow face, retrognathia were present. Severe hypotonia and respiratory insufficiency were dominant signs. MRI of the brain at age two weeks showed no pathological changes. Screening for inherited metabolic disorders was normal. Pompe disease was excluded by screening for the activity of enzyme alpha-glucosidase from the dried blood spot. Levels of creatine kinase were repeatedly in the normal range. The patient had intensive rehabilitation; tracheostomy and percutaneous gastrostomy were performed at three months. At age six months, he underwent a brain MRI with findings of cortico-subcortical atrophy in the frontotemporal region bilaterally, secondary enlargement of ventricles, absence of neurohypophysis signal, mega cisterna magna, cavum veli interpositi, and hypoplasia of the optic nerves bilaterally (**Figure S1A**). Continuing metabolic examination showed no evidence of energetic mitochondrial disorder. There were no signs of cataracts at the age of 2 years. Other genetic causes of floppy infants were excluded according to the in-house approach (see *2. Genetic investigation in P1-P4* below). Whole exome sequencing (WES) focused on congenital myopathies, other neuromuscular disorders, and mitochondrial revealed hemizygous frameshift

variant c.438_439delCA (p.His146Glnfs*10) in exon 6 of the *MTM1* gene. According to the ACMG criteria [18], this variant is pathogenic (class 5 - PVS1+PM2_supp+PP4). The diagnosis of X-linked myotubular myopathy (XLMTM) was established at the patient's age of 3 years and four months. The variant was inherited from an asymptomatic mother in the term of XMTM (**Table 1**). The patient's mother is the carrier of the *de novo* variant in the family. At age six years, P1 is still dependent on ventilator support 24 hours per day and has no independent ambulation. However, he can sit for 30 minutes without support, his motor development progresses without any regression, and his mental development seems to be intact (**Figure S1B**). Next year, he will start regular primary school with an assistant. At the time of article submission, P1 is seven years and three months old. According to [7, 8], the disease phenotype of P1 is classified as a severe (classic) form of XLMTM.

Figure S1. **(A): P1 brain MRI at age six months (picture below):** a) cortico-subcortical atrophy in the frontotemporal region bilaterally with secondary enlargement of the ventricles, mega cisterna magna, and cavum veli interpositi in T2W, b) bilateral hypoplasia of the optic nerves, and c) absence of the neurohypophysis signal in T1, **(B):** Patient 1 (P1) at age six years (picture on the right). P1 with characteristic myopathic phenotype: long face, hypomimia, hypotrophy of the skeletal muscles, muscle weakness, tracheostomy, permanent ventilatory support, minimal spontaneous movements, no ambulation.

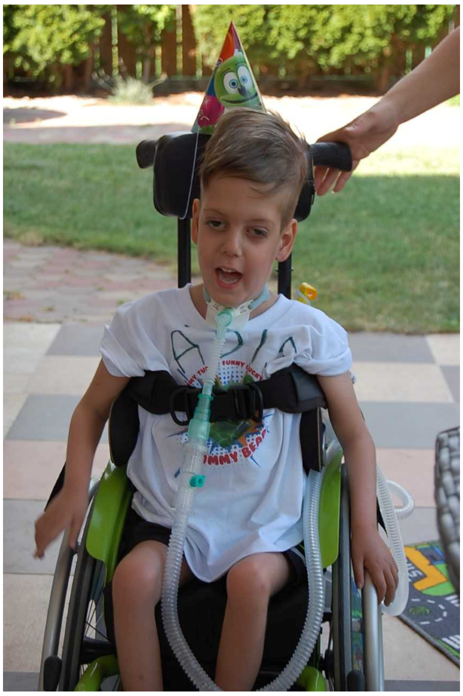

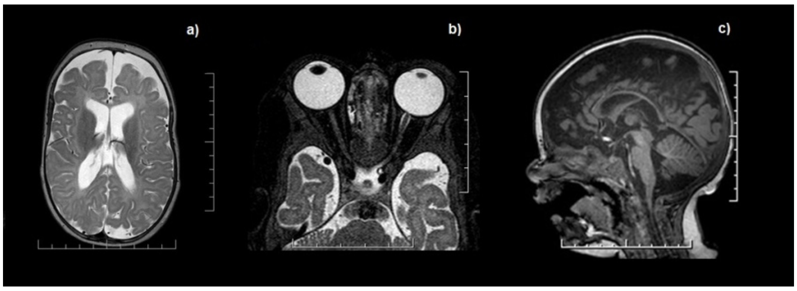

1.2. Patient 2 (P2)

The second patient, P2, is an Austrian (Caucasian) male child of non-consanguineous parents from the mother's first pregnancy. During pregnancy, polyhydramnios, as well as weak fetal movements, were observed. P2 was born spontaneously in 34+4 weeks of gestation; birth weight was 2,740 g (Fenton: 75th percentile), body length was 53 cm (Fenton: > 97th percentile), and head circumference was 33 cm (Fenton: 75th percentile). Joint contractures were present on the upper and lower extremities. The Apgar score was 5 (in the 1st minute), 5 (in the 5th minute) and 7 (in the 10th minute). Immediately after birth, P2 was severely hypotonic (floppy infant) and developed asphyxia with an early need for intubation and respiratory support. P2 had a long face, prominent cheeks, and coarse facial features, and bilateral cryptorchidism was present. Neurologic examination showed, besides severe global hypotonia, weak swallow reflex, weak deep tendon reflexes, and a weak cry. Brain ultrasonography showed dilatation of the ventricles, creatine kinase level was average, and metabolic screening showed no signs of an inherited metabolic disorder. Other genetic causes of floppy infants were excluded according to the in-house approach (see *2. Genetic investigation in P1-P4* below). Investigation by whole exome sequencing was indicated. At the age of three weeks, a muscle biopsy in general anesthesia was performed from the right vastus lateralis muscle, and the result in the next three weeks showed signs of centronuclear myopathy (**Figure S2A, C, and E**). Results from WES confirmed the clinical diagnosis of centronuclear myopathy, concretely the XLMTM in the patient age of 2 months. In the *MTM1* gene, a hemizygous variant c.(342+1_343-1)_(444+1_445-1)del; p.(Asp115_Leu148del) which represents an in-frame exon 6 deletion, was found. According to the ACMG criteria [18], this variant is likely pathogenic (class 4). The variant was inherited from an asymptomatic mother (**Table 1**). At the age of 2 months, tracheostomy and percutaneous gastrostomy were made. MRI of the brain at age three months found hydrocephalus of unknown origin without diapedesis of the cerebrospinal fluid. A ventriculoperitoneal shunt was implanted. An ophthalmologic examination revealed a subcapsular cataract bilaterally. At the time of article submission, P2 is 26 months old, with dominant generalized hypotonia and arreflexia. He can move his hands and feet, turn his head to the sides, and still depend on ventilator support for 21 hours daily. Typical facial and skeletal features of XLMTM are present (**Figure S3**). According to (Herman, 1999; McEntagart, 2002), the disease phenotype of P2 is classified as a severe (classic) form of XLMTM.

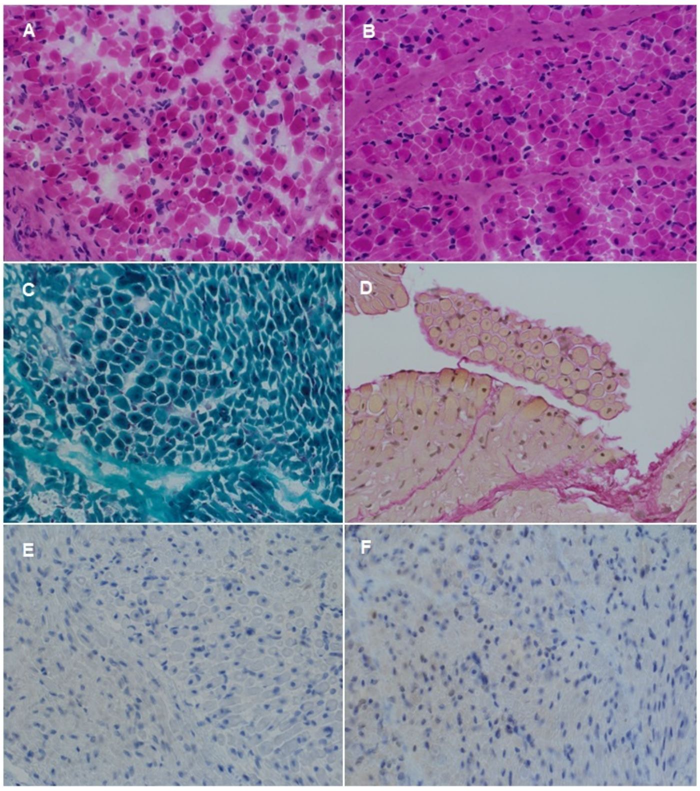

Figure S2. Muscle biopsy – histopathological findings consistent with centronuclear myopathy. Stained sections of the vastus lateralis muscle from Patient 2 (P2) and Patient 3(P3) at the age of three weeks. **(A): P2** and **(B): P3**: hematoxylin and eosin (H&E) numerous small hypotrophic rounded myofibers with varying percentages of centrally located nuclei; **(C): P2**: Trichrome Gomori; **(D): P3**: Elastica van Gieson; **(E): P2** and **(F): P3**: immunohistochemistry: myotubularin is absent.

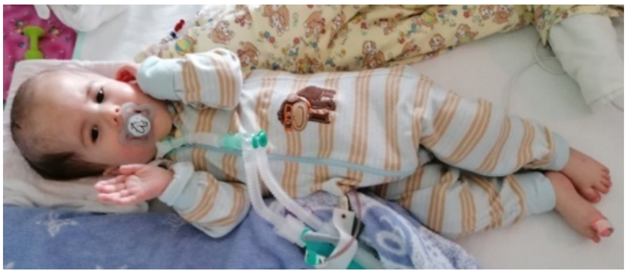

Figure S3. Patient 2 (P2) phenotype features at age eight months. Long, narrow face and figure, fingers of the hands and feet, fatigue appearance, dolichocephaly, global hypotonia, and respiratory insufficiency (tracheostomy with permanent need of artificial ventilatory support).

1.3 Patients 3 and 4 (P3 and P4)

The third patient, P3, was an Austrian (Caucasian) male child of non-consanguineous parents from the mother's third pregnancy complicated with polyhydramnios and weak fetal movements. The first child was a healthy female child, and the second was a male child from risk pregnancy (non-immune hydrops fetalis) who died on the fifth day of life due to respiratory failure (see P4 below). P3 was born in the 37th week of pregnancy by cesarean section and vacuum extraction. Birth weight was 3,320 g (Fenton: 75-90th percentile), body length was 53 cm (Fenton: 97th percentile), and head circumference was 36.5 cm (Fenton: > 97th percentile). Severe global hypotonia (*floppy infant*) and no spontaneous respiratory activity were present immediately after birth, without any reflexes. The Apgar score was 2 (in the 1st minute), 3 (in the 5th minute) and 4 (in the 10th minute). The patient developed severe dyspnea, had to be intubated in the 12th minute of life, and needed artificial ventilator support. Other phenotype features included weak cry, gothic palate, telecanthus, congenital cataract, long fingers, and bilateral cryptorchidism. Joint contractures were present on the upper and lower extremities (**Figure S4**). Other genetic causes of floppy infants were excluded according to the in-house approach (see *2. Genetic investigation in P1-P4* below). Investigation by whole exome sequencing was indicated. At the age of 3 weeks, a muscle biopsy in general anesthesia was performed from the right vastus lateralis muscle, and the result in the next two weeks showed signs of centronuclear myopathy (**Figure S2B, D, and F**). Unfortunately, the patient died on the 44th day of life due to respiratory failure aggravated by aspiration pneumonia. Results from WES confirmed the clinical diagnosis of XLMTM post-mortem (in patient hypothetical age seven weeks). In the *MTM1* gene, a hemizygous variant c.(1053+1_1054-1)_(1467+1_1468-1)del; p.(Leu352_Gln489)del, which represents an in-frame exon 11-13 deletion, was found. According to the ACMG criteria [18], this variant is likely pathogenic (class 4). The variant was inherited from an asymptomatic mother (**Table 1**). We looked retrospectively at the clinical reports of the P3 brother, who was the second child from the second-risk pregnancy of the mother complicated with polyhydramnios, weak fetal movements, and non-immune hydrops fetalis. P4 was born in 36+9 weeks of pregnancy by cesarean section. Birth weight was 3,320 g (Fenton: 75th percentile), body length was 52 cm (Fenton: 90-97th percentile), and head circumference was 36.5 cm (Fenton: > 97th percentile). Asphyxia occurred immediately after birth (pH from the umbilical artery was 6.81). The Apgar score was 2 (in the 1st minute), 2 (in the 5th minute) and 3 (in the 10th minute). Intubation and artificial lung ventilation were required (**Figure S4**). Due to hypoxic encephalopathy, controlled hypothermia was indicated

in the patient for the first three days. Unfortunately, the patient died on day five due to asphyxia. A genetic investigation found normal male karyotype 46, XY. SNP array (Illumina Cyto850Kv1.2 Beatchip) found deletion arr[GRCh37] 10q11.22q11.23 (46,283,686-51,833,731)x1 (length 5,6 Mb). Later, the SNP array analysis in the parents found the same deletion 10q11.22q11.23 in the healthy father. We reevaluated the patient's clinical features, which fulfill the features of XLMTM. The hemizygous variant c.(1053+1_1054-1)_(1467+1_1468-1)del; p.(Leu352_Gln489)del, previously found in the brother (P3) and healthy mother, was proved post mortem by MLPA analysis in the archived DNA of P4.

Figure S4. Patient 3 (P3, on the left) and Patient 4 (P4, on the right) in the neonatal period. Severe myopathic phenotype with permanent ventilatory support need is present in both.

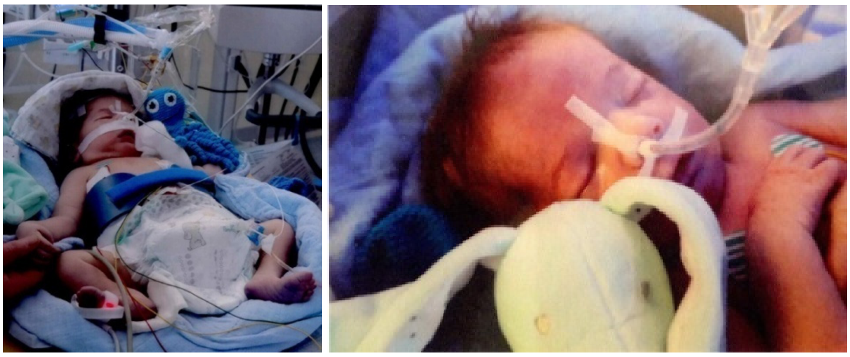

2. Genetic investigation in P1-P4

P1: WES in P1 was performed in BGI, Hong Kong (Complete Genomics Human 59M Exome). Called variants were loaded into the in-house Gemini SQLite database [38] and annotated. Private and rare variants with minor allele frequency ≤0.01 (gnomAD v2.0) were screened for a virtual panel of genes associated with neuromuscular disorders based on [39]. The candidate variant in the *MTM1* gene (NM_000252.2) was verified using Sanger sequencing in the patient and his family members.

P2: Exome sequencing: Enrichment of all exonic DNA fragments using Twist Comprehensive Exome + Mitochondrial Panel (Twist Bioscience). Sequencing on NextSeq2000 (Illumina) as 2x150 bp "paired end reads". Automatic arrangement of the sequences against the human reference sequence GRCh37 (hg19) and data analysis (including copy number changes) with

SegNext (JSI). Analysis criteria: Evaluation of the *MTM1* gene (NM_000252.3) Recording of the coding and flanking intronic sequences (-15/+5) with a targeted reading depth (coverage) of at least 20x. Variants of unclear significance (class 3) and disease-relevant mutations (classes 4 and 5 of the 5-class system were classified and reported according to Plon et al., 2008 [40]. The classification of the variants follows the consensus recommendations of the American College of Medical Genetics [18]. The A in the start codon corresponds to c.1 [41]. Filter criteria: (1) MAF below 1%, (2) variant frequency in reads > 20%, (3) stop, missense and frameshift variants as well as variants in canonical splices.

P3: Exome sequencing: Enrichment of all exonic DNA fragments using the Twist Comprehensive Exome + Mitochondrial Panel (Twist Bioscience). Sequencing on NextSeq2000 (Illumina) as 2x150 bp "paired-end reads", automatic arrangement of the sequences against the human reference sequence GRCh37 (hg19), and data analysis/vcf-file with SeqNext (version 5.0; JSI). Variant analysis: evaluation of the cell genes (including changes in the number of copies with SegNext (JSI)): *BIN1* (NM_139343.3), *DNM2* (NM_001005360.2), *MTM1* (NM_000252.3), *MTMR14* (NM_001077525.3), *RYR1* (NM.000540.2). Detection of the coding and flanking intronic sequences (-15/+5) with a targeted reading depth (coverage) of at least 20x. The findings report variants of unclear significance (class 3) and definitely disease-relevant mutations (classes 4 and 5 of the 5-class system according to Plon et al., 2008 [40]. The classification of the variants follows the consensus recommendations of the American College of Medical Genetics [18]. The A in the start codon corresponds to c.1 [41]. Heterozygous gene mutations for autosomal recessive diseases that could mean carriers are not reported. Filter criteria for variant search: (1) MAF < 1%, (2) coverage ≥ 20-fold, (3) variant frequency in reads > 30%, (4) stop, missense, and frameshift variants as well as variants in canonical splices, (5) not classified as benign/probably benign in ClinVar.

3. Additional studies to confirm the pathogenicity of novel variants

3.1. Methods

3.1.1 Muscle biopsy

The vastus lateralis muscle biopsy was performed in P2 and P3 under general anesthesia according to standard procedures. Muscle tissue was cut into several pieces for formalin fixation, glutaraldehyde fixation, and rapid freezing in isopentane cooled in liquid nitrogen for subsequent analyses [42].

3.1.1.1. Histopathology

The histopathology and the enzyme histochemical stain analyses encompassed the following stains: Haematoxylin and Eosin (H&E), Elastica van Gieson (EvG), Congored, ATPase at pH 9,4, pH 4,3 and 4,2, reduced nicotinamide adenine dinucleotide-tetrazolium reductase (NADH), Gomori trichrome, cytochrome c oxidase (COX), succinate dehydrogenase (SDH), acid phosphatase, periodic acid-Schiff (PAS), Sudan black, myoadenylate deaminase (MAD), phosphofructokinase (PFK), phosphorylase, and acetylcholinesterase (ACE). The immunohistochemical analyses included the following antibodies: CD3, CD20, CD8, CD4, CD45, CD79a, CD68, HLADRII, beta-spectrin, alpha-sarcoglycan, beta-sarcoglycan, gamma-sarcoglycan, delta-sarcoglycan, dystrophin (N-terminus), dystrophin (C-terminus), dystrophin (rod domain), dysferlin, titin, emerin, telethonin, POMT1, myotilin, lamin A/C, caveolin-3, actinin, laminin2, collagen VI, desmin, myosin fast, myosin slow, myosin neonatal, membrane attack complex, vimentin, and utrophin at appropriate dilutions.

3.1.1.2. Immunohistochemistry

Immunohistochemistry was performed on formalin-fixed and paraffin-embedded (FFPE), 5μm-thick sections on Superfrost Plus Adhesion Microscope slides (J1800AMN2, Epredia, USA). Deparaffinized, rehydrated sections underwent antigen retrieval in the PT Module (Epredia, USA) using the Dewax and HIER Buffer L (pH XY) (Epredia, The Netherlands).All subsequent steps were carried out using the Autostainer 480 (Epredia, USA) and the UltravisionQuanto Detection System HRP (TL-125-QHL, Epredia, The Netherlands).The reaction product was visualised using diaminobenzidine chromogen (DAB Quanto, TA-125-QDX, Epredia, and The Netherlands). Then, the sections were counterstained with Mayer'shematoxylin (Thermo Scientific,

UK).Antibodies were diluted using the AB diluent OP Quanto (ready-to-use) TA-125-ADQ (Epredia, The Netherlands). As negative control, the primary antibody was omitted and was replaced by normal mouse serum. The characteristics of the used primary antibody are as follows: myotubularin (clone Y290), dilution 1:50, Biozol, Germany.

3.2. Results

3.2.1. Histopathology

The light microscopic examination of the vastus lateralis muscle from P2 and P3, performed in both patients at the age of 3 weeks, revealed the typical picture of centronuclear myopathy, i.e., numerous small hypotrophic rounded myofibers with varying percentages of centrally located nuclei (**Figure S2**).

3.2.2. Immunohistochemistry

The immunohistochemical analysis showed a complete loss of myotubularin in the biopsy tissue from **P2** and **P3**. Myotubularin could be demonstrated in muscle biopsies from controls, i.e., patients with other neuromuscular disorders (**Figure S3**).
